# The Montreal Veterinary Medical Association

**Published:** 1895-03

**Authors:** E. H. Lehnert

**Affiliations:** Secretary-Treasurer


Secretary.

### THE MONTREAL VETERINARY MEDICAL ASSOCIATION.

The fourth meeting of the session, 1894–95, of the Montreal Veterinary Medical Association was held in the lecture-room of the Faculty of Comparative Medicine, on Thursday evening, November 29th, with the President, Dr. Adami, in the chair. Drs. Baker and Dawes were also present.

After the roll-call, reading and adoption of the minutes of the previous meeting, the report of the Experiment Committee was received. Mr. Dell then called the attention of the Society to the fact that while reports of these meetings were furnished the local newspapers, the Reporting Committee had, owing to pressure of other work, been unable to furnish the various journals with reports, and suggested that type-written reports be sent to the veterinary journals.

The President expressed himself in favor of sending reports of original work, experimental or otherwise, to the journals. It would prove a stimulus to the essayists to do more work of this nature. It was decided to leave this to be carried out by the Reporting Committee.

Mr. C. H. Zink presented the Association with the details of an operation for the removal of a tumor from the vagina of an aged cocker spaniel bitch, the latter and also the tumor being exhibited to the members after the paper had been read. Dr. Adami had microscopically examined the tumor and found it to be of myosarcomatous nature evidently originating from the muscular tissue of the uterus.

These tumors are frequent in the human subject, and may develop in three ways, viz., under the serous coat (subserous), in the uterine wall (intramural), or under the mucous coat (submucous).

The Doctor illustrated his remarks in a very instructive manner by blackboard diagrams.

Mr. J. C. Hargrave read a paper on "Canine Distemper," presenting a *résumé* of the history, nature, causes, symptoms, complications, and treatment of this disease, special reference being made to the work of Mr. Everett Millais, who claims to have discovered the specific germ of the disease and produced immunity by inoculation.

A short discussion followed, Dr. Adami stating that the work of Mr. Millais had not been confirmed by recent observations and that the germ of distemper still remained undiscovered. He pointed out that Dr. Cecil French, working in his laboratory last year, had apparently repeated Millais' observations, staining a micro-organism resembling his, which, however, was lost or disappeared during the course of inoculation.

The meeting then adjourned.

The Montreal Veterinary Medical Association met in the lecture-room of the Faculty of Comparative Medicine on Thursday evening, December 13, 1894, with the President, Dr. J. G. Adami, in the chair. Professors D. McEachran and Baker were also present. After roll-call the minutes of the last meeting were read and adopted.

A letter was read from Dr. Dinwiddie donating a copy of his work on micro-biology to the library. The Secretary was instructed to convey the thanks of the Association to the donor.

On suggestion of Mr. Thurston, an additional day was appointed by the Chair on which members can remove books from the library, but it is the privilege of the members to consult the books at any time during the day.

Messrs. Clark and Boutelle reported on behalf of the Experiment Committee the experiments with anæsthetics.

Mr. E. H. Lehnert read notes on a case of protrusion of the rectum in a dog, describing the method of removal by sutures which, by constriction, produced sloughing of the everted portion.

Dr. Adami suggested that blunt scissors, so useful in the removal of hemorrhoids, might also be used to advantage in these cases.

Mr. Parker stated that he had used blunt scissors in castrating pigs, with excellent results.

At this juncture Dr. Adami was called away, but before expressing his regrets he informed the Society that he would exhibit a "Murphy button" at the next meeting, and explain the indications and methods for its use.

The Honorary President, Dr. D. McEachran, occupied the chair during the remainder of the evening.

Mr. C. A. Boutelle read a paper on the subject of "Enchondroma," giving a brief *résumé* of the work of German and English writers on the subject, and adducing the results of some original investigations in the pathological laboratory of the University. He described the microscopic appearance of a tumor, weighing over ten pounds, found in the abdomen of a spaniel bitch on post-mortem. This was secondary to an enchondroma of the mammary gland removed a year previously.

The cartilaginous matrix of this growth had in some portions been replaced by a mucoid, gelatinous material, and in others undergone calcification. Fibrous tissue was also present, and in the peripheral region was a considerable number of fat cells. Such a variety of tissue had, in fact, been found that the essayist described the growth as being of lipo-myxo-osteo-sarcomatous nature.

In the lungs and in some of the lymphatics were found growths having a tendency to osteoid formations. Another mass found also in the mammary gland, but at the site of the original operation, conformed in appearance to what human pathologists would term either malignant adenoma or carcinoma.

Dr. D. McEachran referred to the paucity of literature on the subject. He had, however, seen similar cases, and found in nearly all a tendency to ossification.

After further discussion, the Chair appointed Messrs. Cowan and Inglis as essayists for the next meeting.

The meeting then adjourned.

The Montreal Veterinary Medical Association met in the college lecture-room January 25, 1895, with the President, Dr. Adami, in the chair. Drs. Baker and Gunn were also present.

After the reading and adoption of the minutes of the previous meeting, Mr. W. K. Inglis presented the report of the Experimental Committee. The work had consisted of experiments demonstrating the action of alcohol and nicotine on the amphibian heart. Small doses of the former stimulated, while larger doses depressed the action of the heart muscle. The latter was depressing throughout, the depression being in proportion to the dose.

Dr. Adami pointed out the extreme irregularity of the frog's heart in its reaction to drugs—irregularity depending on period of year, sex, age, etc., so that as he personally had experimented, it becomes hopeless to make exact parallel observations and to arrive at any definite conclusions.

The Secretary read a communication from Dr. McIntyre in regard to his experience with hypodermatic injections of large doses (gr. x) of mor. sulph. in the treatment of colic.

The letter evoked considerable discussion on the part of Drs. Adami,

Baker, and Gunn, it being the consensus of opinion that with smaller doses the treatment would not have proved so unsuccessful.

Mr. W. V. Jones read notes on a case of purpura hemorrhagica. The treatment consisted of intertracheal injections of an aqueous solution of iodine and pot. iodi., but as the case terminated fatally the merits of this treatment from this one case could not be determined.

Mr. J. C. Cutting presented a carefully prepared paper on "Tuberculosis, its Sanitary Aspects and Relations to Animal Industry." The subject was thoroughly treated, particular stress being laid on prophylactic measures, proper feeding, housing, and the abolition of inbreeding. The value of tuberculin as a diagnostic agent was also referred to.

A brief but spirited discussion followed, after which the meeting adjourned.

The Montreal Veterinary Medical Association met in the lecture-room of the Faculty of Comparative Medicine on February 7, 1895, the President, Dr. Adami, occupying the chair. Minutes of previous meeting were read and adopted.

Mr. E. H. Lehnert reported an experiment with morph. sulph. as an aid to the production of chloroform anæsthesia in the horse. The subject, an aged animal, had received an intravenous injection of the sulphate of morphine (grs. v, in solution), and subsequently it was found that chloroform ℥ij was sufficient to induce complete anæsthesia.

Mr. W. K. Inglis read an instructive paper on the subject of "Anthrax," describing the geographical distribution, etiology, symptoms, pathology, and treatment of the disease.

The paper elicited an animated discussion from those present, Dr. Baker stating that the disease was of comparatively rare occurrence in this Province (Quebec), and the cases which he had witnessed were in such an advanced stage as not to prove amenable to treatment. In one case the disease appeared among cattle confined in a field in which diseased animals had been buried.

Dr. Adami, referring to other outbreaks of the disease in Ontario, said that these had occurred in the vicinity of tan-pits, where hides had been brought from other places.

Handling of diseased skins is also the medium of contagion in "wool-sorter's" disease and carbuncle. He advised inoculation in the case of anthrax as a prophylactic measure, when the disease exists over a large area, and in restricted outbreaks the keeping of animals off fields in which diseased ones have been buried. He also advised great care in holding post-mortems, and pointed out the dangers to the veterinarian which attend the same.

Mr. E. C. Thurston read a paper on "Modes of Restraint and Anæsthesia," but owing to the lateness of hour its discussion was deferred.

The meeting then adjourned.

E. H. Lehnert,
Secretary-Treasurer.

